# Whole Exome Sequencing Identified a Stop-Gained Mutation in DYSF Gene Associated With Dysferlinopathy in an Iranian Family

**DOI:** 10.1155/ijog/9103068

**Published:** 2025-07-23

**Authors:** Saba Baghshomali, Asiyeh Jebelli, Mina Kazemzadeh, Farzaneh Jalalypour, Mohammad Yazdchi, Amir Ali Mokhtarzadeh, Leila Emrahi

**Affiliations:** ^1^Department of Biological Sciences, Faculty of Basic Sciences, Higher Education Institute of Rab-Rashid, Tabriz, Iran; ^2^Department of Cell and Molecular Biology, Faculty of Biological Sciences, Kharazmi University, Tehran, Iran; ^3^Department of Animal Biology, Faculty of Natural Sciences, University of Tabriz, Tabriz, Iran; ^4^Department of Computer Science and Engineering, Section for Data Science and AI, Chalmers University of Technology, Chalmersplatsen 4, Gothenburg, 412 96, Sweden; ^5^Neurosciences Research Center, Tabriz University of Medical Sciences, Tabriz, Iran; ^6^Immunology Research Center, Tabriz University of Medical Sciences, Tabriz, Iran; ^7^Department of Medical Genetics, Faculty of Medical Sciences, Tarbiat Modares University, Tehran, Iran

**Keywords:** *DYSF*, dysferlin, LGMD, muscular dystrophy, WES

## Abstract

**Introduction:** Muscular dystrophy (MD) refers to a group of hereditary disorders characterized by progressive muscle degeneration, often caused by a deficiency or insufficient levels of glycoproteins in muscle cell membranes. Mutations in various genes lead to different types of MD, each with distinct clinical manifestations and inheritance patterns. The genetic heterogeneity of MD complicates the identification of the causative genes.

**Materials and Methods:** This research was conducted to identify the genetic basis of MD in an Iranian family with three affected members. Whole exome sequencing (WES) was performed on a proband who had initially been misdiagnosed with polymyositis. Following the identification of the disease-causing variant via WES, cosegregation analysis was carried out among two affected siblings, the asymptomatic parents, and one unaffected sibling.

**Results:** WES identified a homozygous nonsense variant (c.6001C>T, p.Gln2001Ter) in Exon 53 of the *DYSF* gene, which encodes dysferlin, a transmembrane protein essential for membrane protection and repair following damage. This stop-gain mutation results in a nonfunctional truncated protein lacking the transmembrane helix, preventing its anchorage to the membrane. Dysfunction of dysferlin is associated with limb–girdle muscular dystrophy 2B (LGMD2B) and Miyoshi myopathy.

**Discussion:** Bioinformatics analyses and clinical findings confirmed the pathogenicity of this variant in a homozygous state, consistent with autosomal recessive inheritance. Furthermore, structural modeling suggested that the mutation significantly disrupts the tertiary structure of dysferlin. Since the disorder onset in the proband and his two affected sisters began in the proximal limb muscles, the condition was classified as LGMD. The study highlights the diagnostic value of WES in accurately identifying disease-causing variants, offering substantial improvements in time and cost efficiency over conventional diagnostic procedures.

## 1. Introduction

Muscular dystrophy (MD) refers to a heterogeneous group of hereditary disorders characterized by progressive muscle atrophy, resulting from the deficiency or absence of essential proteins in the muscle plasma membrane, or dysfunction of key proteins to maintain the structure and integrity of muscle cells [1, 2]. This condition leads to the deterioration of muscle cell integrity, causing severe pain, mobility impairment, and mortality due to cardiorespiratory failure [3]. MD exhibits clinical, genetic, and molecular heterogeneity [4]. It is caused by mutations in multiple genes and follows various modes of inheritance, including autosomal dominant, autosomal recessive, and X-linked patterns [3, 5]. These genes influence the inheritance pattern, age of onset, symptom severity, disease progression, and involvement of the respiratory and cardiac systems [6, 7]. While some MD subtypes primarily affect skeletal muscles, others also impact cardiac and respiratory muscles, which can be life threatening [7]. Secondary pathological features, such as inflammation, oxidative stress, sarcolemmal instability, calcium influx, extracellular matrix degradation, and fiber necrosis, emerge as downstream consequences of the primary deficiencies [8, 9].

The most common types of MD are Duchenne dystrophy, Becker dystrophy, myotonic dystrophy, facioscapulohumeral dystrophy, limb–girdle muscular dystrophy, oculopharyngeal dystrophy, Emery–Dreifuss dystrophy, and Miyoshi myopathy [7]. Some MDs are classified into subgroups. Among them, limb–girdle dystrophy encompasses the widest range, with more than 30 different types identified [7, 10, 11]. Despite the fact that some phenotypic features are specific to certain MD types, many similar clinical characteristics—including difficulty in physical activity and performing daily tasks—overlap, leading to nonallelic heterogeneity [7]. In addition, nongenetic factors such as viruses or toxins that affect the nervous system can imitate the MD phenotype. Therefore, precise distinguishing phenocopies and validating candidate genes and mutations are essential for achieving an accurate diagnosis [12].

Dysferlinopathy refers to a clinically heterogeneous group of MD inherited in an autosomal recessive pattern. The two major phenotypes of dysferlinopathy are limb–girdle muscular dystrophy Type 2B (LGMD2B) and Miyoshi muscular dystrophy (MMD), while distal myopathy with anterior tibial onset (DMAT) and asymptomatic hyperCKemia are considered minor phenotypes [12–14]. LGMD2B has been renamed “limb–girdle muscular dystrophy R2” (LGMDR2) dysferlin related and primarily impacts the proximal muscles, particularly those in the thighs. In contrast, MMD1 predominantly targets the distal muscles, especially the calves. DMAT is characterized by early and pronounced weakness in distal muscles, particularly in the anterior muscles of the legs. The hyperCKemia is marked by a notable rise in serum creatine kinase (CK) levels without any accompanying clinical symptoms [13]. To date, there is no approved cure for dysferlinopathy. Management is supportive and is aimed at reducing disease-related complications through interventions such as mobility aids, physical therapy, respiratory support, and orthopedic surgery when necessary [15]. Dysferlinopathy caused by mutations in the *DYSF* gene is located on the short arm of Chromosome 2 (2p13) [16]. This gene comprises 58 exons (Gene ID 8291, NCBI database) and extends approximately 150 kb of genomic region. *DYSF* encodes a 230 kDa protein called dysferlin, a membrane-anchored protein that belongs to the ferlin family. Dysferlin is found in various tissues, with particularly robust expression in the brain, placenta, skeletal, and cardiac muscles [17, 18]. In mature myofibers of skeletal muscles, *dysferlin* is mainly localized to the muscle plasma membrane (sarcolemma), but it is also present in T-tubule and cytoplasmic vesicles [19, 20]. Both pathogenic and likely pathogenic mutations discovered in the *DYSF* gene are typically point mutations or small indel mutations [21]. While many of these mutations are nonsense that result in truncated, nonfunctional dysferlin protein, missense mutations have also been reported throughout the *DYSF* gene. Identified mutations may result in partial or complete loss of dysferlin function [22]. Due to the clinical heterogeneity of dysferlinopathy, identical mutations in the *DYSF* gene can give rise to different phenotypes, including LGMDR2 and MMD, within the same family or may manifest as overlapping phenotypes in an individual patient [21].

Considering the heterogeneity of MD, identifying the involved genes and the disease-causing mutations can be challenging, costly, and time-consuming when using conventional techniques such as polymerase chain reaction (PCR) and Sanger sequencing. Next-generation sequencing (NGS) has revolutionized the approach to identifying genes implicated in common, rare, and heterogeneous disorders. It facilitates the comprehensive detection of both known and de novo mutations, without prior assumptions about specific genes [23]. NGS is a high-throughput technique that allows the rapid sequencing of large amounts of DNA or RNA and is classified into three main types: whole genome sequencing (WGS), whole exome sequencing [24], and targeted gene panel [25, 26]. Among these, WES is particularly valuable as it sequences all protein-coding regions within a genome, promoting the identification of disease-causing variants in a patient's DNA [27].

The present study was conducted to investigate an Iranian family with three members affected by myopathy, utilizing WES to enhance our understanding of the genetic changes associated with this disorder.

## 2. Materials and Methods

### 2.1. Patients

**Figure 1:**
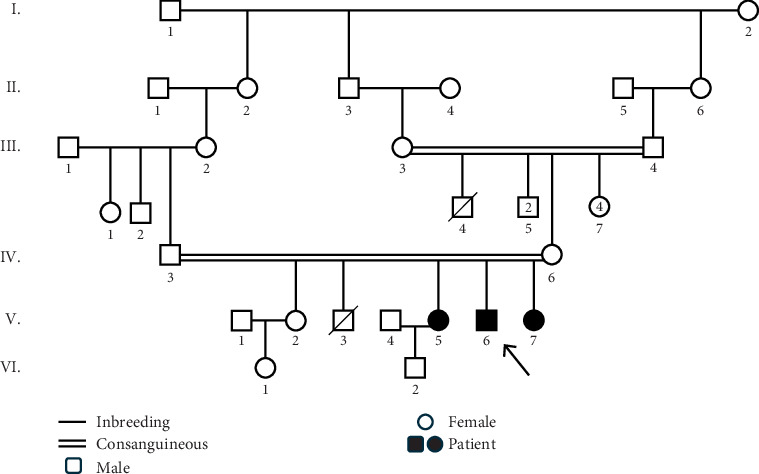
The pedigree of a family with three members diagnosed with myopathy. The filled black symbols represent affected individuals. The white symbols indicate members without any myopathy-related symptoms. The arrow indicates the proband of the family. Doubled lines show the occurrence of consanguineous marriage.

This research focused on a family with three members experiencing muscle weakness, one son (proband) and two daughters. Initially, the proband's condition was misdiagnosed as “polymyositis” based on a muscle biopsy. However, after 3 months of medication, the symptoms worsened. The family was referred to the neurology department at Imam Reza Hospital in Tabriz. Their condition was clinically diagnosed as myopathy. This diagnosis was made based on a thorough clinical assessment performed by experienced neurologists, alongside muscle biopsies of the affected individuals. The family was referred to the Genetic Diagnosis Center for an accurate diagnosis of the myopathy type and the involved genes. Genetic counseling was conducted, and the family pedigree was created (Figure 1). The study received approval from the ethical review committee at Imam Reza Hospital in Tabriz (IR.TABRIZU.REC.1402.029). Informed consent was obtained from all participants in this study, and the study adhered to the principles outlined in the Declaration of Helsinki.

In this study, the parents were in a consanguineous marriage and had four children, one son and three daughters. The son and the two daughters exhibited symptoms of myopathy. The parents and one of the daughters did not show any symptoms related to myopathy. Additionally, the parents had a son who had passed away at the age of 9 due to a seizure. WES was performed on the proband, and a cosegregation analysis was conducted for the other affected and unaffected family members.

#### 2.1.1. Case V.6

The proband was a 27-year-old man who had been experiencing symptoms related to a decrease in physical strength for 10 years. Initially, the disease started in the lower limbs, and the problem was more related to rising from the ground and climbing stairs. The right side of the body was more involved. Gradually, with the disease's progression, walking became impaired, and he had to use a wheelchair. The muscle strength of lower extremities proximal and distal was 2/5 (Medical Research Council [MRC] Scale), while the proximal and distal upper extremities were reported as 2.5 and 3/5, respectively. The Modified Rankin Scale (MRS) of this case was 4. The concentration of serum CK indicated a strong increase of 4132 U/L (reference range: 24.0–170.0 U/L); deep tendon reflexes (DTRs) of the upper and lower limbs were absent. Echo, mental activity, and eye movements were normal, and no signs of diplopia, ptosis, ophthalmoplegia, or nystagmus were observed. Swallow, breath, and speech were normal. The patient did not have any fasciculation. The sensory exam, including superficial touch, pain, temperature, proprioception, and vibration sensation, was normal. Muscle biopsy results indicated fiber size variation with the presence of basophilic fibers and possible fibrosis. Also, nerve conduction studies (NCSs) showed some compound motor action potential (CMAP) with mildly low amplitude. Other NCS and sensory nerve action potentials (SNAPs) were normal. Myogenic changes were observed in some of the sampled muscles.

#### 2.1.2. Case V.5

The patient was a 29-year-old woman with a 14-year history of aggravated weakness of the lower limbs, which started with difficulty in getting up from the ground and climbing stairs. As the disease progressed, she needed help walking. However, physiotherapy exercises improved her ability to walk without any assistance. The muscle strength of the proximal and distal muscles was 3/5. Upper extremities were proximal 3/5 and distal 4/5. MRS was 3. The serum CK level was elevated to 931 U/L (reference range: 24.0–195.0 U/L).

#### 2.1.3. Case V.7

The patient was a 24-year-old woman with decreased strength in the leg muscles starting at the age of 19. Compared with other affected family members, she showed milder symptoms. She had difficulties in bending and straightening and needed help getting from the ground. The muscle strength of upper extremities proximal and distal was 4/5; the muscle strength of lower extremities was proximal 4/5 negative and distal 4/5. MRS was 2. She had a high level of CK (463 U/L, reference range: 24.0–195.0 U/L) and walked with a limp.

### 2.2. WES Technique

After elucidation the rationale behind conducting the examination and obtaining the necessary approval from the subjects, blood specimens were collected. Genomic DNA was extracted from proband's peripheral blood using the salting-out method [28]. The quality and quantity of DNA were evaluated by 1% agarose gel and a NanoDrop 1000 Spectrophotometer, respectively. The extracted DNA was randomly fragmented into 180–280 bp segments, and DNA libraries were prepared by end repair, A-tailing, and adapter ligation. Library enrichment was conducted using SureSelectXT Human All Exon V6. The enriched libraries were sequenced on the Illumina HiSeq 4000 system at CeGaT GmbH Germany with an average coverage of 100× [29].

The coding and flanking intronic regions were enriched and sequenced using Agilent's in-solution technology. First, the raw sequencing data was checked for quality using two industry-standard tools: FastQC and Trimmomatic to clean up the data by trimming low-quality sections [30]. Next, the processed DNA sequences were mapped to the latest human reference genome GRCh37 using the Burrows–Wheeler Aligner software [31], which carefully matches each read to its proper location. After this initial alignment, we refined the results using GATK's tools—adjusting the alignment around tricky insertions/deletions (IndelRealigner) and then fine-tuning the accuracy of individual DNA base calls (BaseRecalibrator) to ensure the most reliable genetic data possible for analysis. Single-nucleotide variants (SNVs) and small insertions or deletions (indels) were identified using HaplotypeCaller of GATK package [32–34].

Variants were filtered by excluding those with a minor allele frequency (MAF) greater than 0.01 using population databases including gnomAD, HapMap, the 1000 Genomes Project (1000G), and Iranom, an Iranian population database comprising 800 Iranian samples from eight different ethnicities. Synonymous variants were excluded, while variants affecting splice site regions were retained for further analysis. This process incorporated multiple databases, including avSNP147, avSNP150, dbNSFP V4.2c, and dbscSNV V1.1, with annotations tracked via the UCSC Genome Browser. We classified genetic variants according to the guidelines set by the American College of Medical Genetics and Genomics (ACMG), sorting them into groups that included harmful (pathogenic), probably harmful (likely pathogenic), uncertain (variants of uncertain significance or VUS), probably harmless (likely benign), and harmless (benign) categories. To better understand how potentially harmful variants might affect health, we used the OMIM database and reviewed relevant medical literature to see how these changes related to specific symptoms or diseases. For those variants that stood out as being especially interesting or important, we went a step further by examining family members—both those with and without the condition—to see if the variant appeared more often in affected individuals, which helped us connect the genetic change to the clinical picture.

### 2.3. Sanger Sequencing Validation and Cosegregation Analysis

Sanger sequencing was accomplished in the proband, affected siblings, parents, and unaffected healthy sister to validate the pathogenic variant and track cosegregation patterns. The suspected disease-causing variant in Exon 53 of the *DYSF* gene was amplified using the following primers F: 5⁣′-AGTGATCGAGAAACCCTTGGC-3⁣′ and R: 5⁣′-TGGGAGGAAAGAGGGAGAATGC-3⁣′ by PCR on a SimpliAmp Thermal Cycler (Thermo Fisher Scientific). PCR product sequencing was performed on an ABI 3500xL PE Sequencer (Applied Biosystems) following the Sanger method, and the results were analyzed using the ChromasPro V2.1.3 software.

### 2.4. Model Building

**Figure 2:**
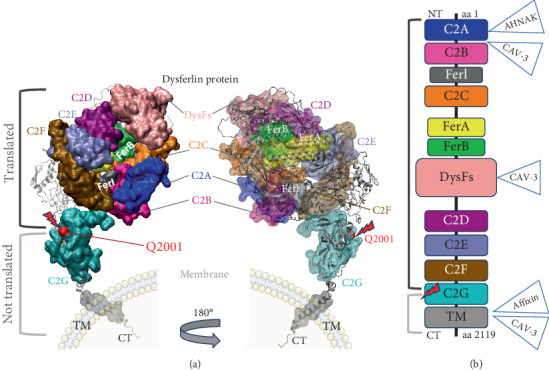
The tertiary structure of the dysferlin transmembrane protein, illustrating the spatial arrangement of its domains. (a) The figure highlights the location of a mutation within the C2 domain. (b) The functional domains of dysferlin are arranged from the N-terminal to the C-terminal as follows: seven C2 domains, three FerA domains, and one DysF domain. The detected mutation is located in the C2G domain near the C-terminal. The structure was predicted using AlphaFold and visualized with the VMD tool [38].

In the absence of an experimentally determined structure for the full-length dysferlin, we employed AlphaFold3 [35, 36], an AI-based structure prediction tool, to predict the protein 3D structure. We first compared the canonical protein sequences from the UniProt database (dysferlin, O75923) with our variant (Figure S1) using PROMALS3D [37]. Excluding the regions truncated by the premature stop codon, the remaining sequence exhibited high similarity to the canonical dysferlin based on sequence alignment (Figure 2). The canonical sequence was subsequently used to model the complete tertiary structure of the protein and to map the location of the mutation and the missing domains.

## 3. Results

### 3.1. WES Analysis of the Proband

To further assess pathogenicity, we selected all homozygous and heterozygous variants identified after applying filters specific to the myopathy-related gene panels. The variants were individually arranged into one of five pathogenicity groups according to the ACMG guidelines for interpreting sequence variants: Class 1, benign; Class 2, likely benign; Class 3, unknown significance; Class 4, likely pathogenic; and Class 5, pathogenic. The WES data revealed a homozygous variant NP_001124459.1:p.Gln2001Ter c.6001C>T, NM_001130987.2:c.6001C>T (in canonical sequence NP_003485.1:p.Gln1962Ter, c.5884C>T, and NM_003494.4) in Exon 53 of the *DYSF* gene in the proband (V.6). This variant, referenced by SNP Number rs1064794020, is classified as a pathogenic category based on ACMG criteria. The p.Gln2001Ter variant has also been reported in the genome aggregation databases (gnomAD, ExAC, or 1000G database) under the name of 2-71679173 C-T.

### 3.2. Validation and Cosegregation Analysis by Sanger Sequencing

**Figure 3:**
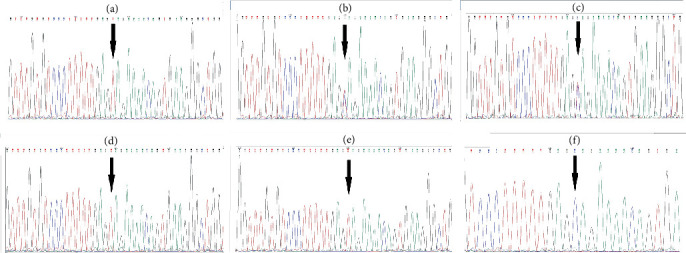
Sanger sequencing validation of c.6001C>T variant discovered by WES in Exon 53 of the *DYSF* gene. The chromatograms indicate the sequences of this variant relative to (a) proband, (b) father, (c) mother, affected sisters (d) V-5 and (e) V-7, and (f) unaffected sister. The position of nucleotide substitution is displaced by an arrow.

To validate the variant reported by WES, direct Sanger sequencing was performed on the proband (Figure 3). For segregation analysis, Sanger sequencing was also conducted for the proband's parents and his siblings (Figures 3, 3, 3, 3, and 3). The findings provide further evidence for the inheritance of this variant within the family and eliminate the possibility of it being a de novo variant. Both parents (IV.3 and IV.6) carry one copy of the variant, but do not exhibit any clinical symptoms. The mutation was identified as a homozygous variant in proband's affected sisters (V-5 and V-7). In contrast, the variant was not present in the proband's healthy sister (V-2) who has not shown any symptoms of MD.

## 4. Discussion

The dysferlin, a large Type II transmembrane (TM) protein, has a complex multiple-domain structure, comprising a small TM sequence near its C-terminal region and a bulk cytoplasmic segment that includes Fer (FerI/A/B) domain, DysF domain, and seven intracellular C2 (C2A–G) domains (Figure 2) that mediate protein-binding and lipid-binding interactions [39]. C2 domains have a beta-sandwich structure, and the binding of two or three calcium ions is usually facilitated by four to five conserved aspartate residues located on the loops at one end of the domain. *C2* domains, which are also found in many other proteins, are distinguished for their calcium-dependent interactions with anionic phospholipids or effector proteins. Calcium significantly increases membrane binding for C2A, C2E, C2F, and C2G more than for C2B and C2D, and all seven domains exhibit some level of membrane-binding affinity even in the absence of calcium [40]. Among all C2 domains of the dysferlin protein, only the canonical C2A domain is known to interact with both calcium ions and lipids [41]. It seems that calcium converts C2A from a flexible, low-affinity state for phosphoinositide phosphatidylinositol 4,5-bisphosphate (PI[4,5]P_2_) to a high-affinity state, enabling dysferlin to localize to PI(4,5)P_2_-enriched membranes through interaction with Tyr23, Lys32, Lys33, and Arg77 [42]. While dysferlin primarily colocalizes with different types of proteins on the sarcolemma and T-tubules in skeletal muscle [43–45], in other cell types and tissues, this protein is found at the plasma membrane and on membrane-bound vesicles related to exocytosis or endocytosis [46]. The more N-terminal C2 domains play a critical function in forming multiprotein complexes with ligands such as TRIM72/MG53 and AHNAK; however, the binding sites of several other protein partners, such as caveolin-3, SNARES, and dihydropyridine receptor, remain unidentified [46].

Membrane repair depends on the gathering and merging of vesicles near the site of membrane damage. Dysferlin, by regulating vesicle trafficking and fusion, probably regulates regeneration or repairing the membrane in muscle cells [45]. It was indicated that sarcolemma damage in dysferlin-null muscle is linked to the buildup of vesicles beneath the membrane [47]. During membrane injury of the muscle cells, dysferlin plays an integral role in a muscle-specific protein complex, responsible for exocytotic repairing of the membrane, where it is associated with effector proteins such as MG53, caveolin-3, annexin, and AHNAK [16]. AHNAK binds to annexins and actin to ensure membrane repair stability and also interacts with C2A, forming a docking site for other proteins involved in membrane regeneration or repair. Dysferlin is essential for cell survival postinjury by patching defects and sealing the membrane breach by orchestrating the vesicles fusion with the sarcolemma at the microlesion site [48]. The evidence proposes that while missense mutations in the C2 domain of dysferlin do not significantly reduce its amount, they diminish the phospholipid-binding activity of dysferlin, leading to its improper localization and an inability to associate with the AHNAK and MG53 proteins [22, 44]. Dysferlin plays a critical role not only in direct muscle repair but also in the immune response to muscle injury, as it is present in the secretory and plasma membrane vesicles of polymorph nuclear neutrophils [49]. Dysferlin misfolding leads to the formation of a cross-linked beta-amyloid structure that stimulates the inflammatory response. Consequently, dysferlin fails to carry out its pivotal task in maintaining muscle structure and repair [22].

The conclusion can be adopted that dysferlin provides its function through (1) interaction sites essential for assembling proteins, (2) a domain sensitive to changes in the amount of calcium, and (3) a binding-related region for localization in the cell membrane. Any alteration in these areas leads to perturbation in the recruitment of proteins, failure to respond to calcium concentration, and disability to be placed in the muscle cell membranes correctly. The overall outcome of these phenomena is the loss of muscle cell membrane integrity, membrane destruction, and an impaired ability to regenerate the damaged membrane.

More than 500 distinct types of mutations have been identified in the *DYSF* gene [50]. Although a few recent studies [21, 51] have suggested a potential hotspot region within the inner DysF domain of the *DYSF* gene, pathogenic mutations are distributed throughout the gene. This widespread distribution makes it difficult to consider a precise, specific hotspot area for clinical diagnostic purposes [50]. Considering that numerous exonic and splicing variants have been reported in the *DYSF* gene, sequencing of all 58 exons of the gene is required, which significantly increases the overall cost. Moreover, the phenotypic similarities among dystrophinopathies can lead to misdiagnosis of the disease and the associated causative gene, resulting in unnecessary delays and increased costs. However, advanced techniques like WES enable comprehensive sequencing and analysis of all coding regions in a patient's genome, offering a more accurate and efficient diagnostic approach.

In the present study, we examined an Iranian family with three members affected by myopathy. Genetic counseling was conducted, and WES was performed for the proband's genome. The WES analysis identified a nonsense variant, p.Gln2001Ter, located in Exon 53 of the *DYSF* gene. While most previously identified pathogenic mutations are located within the DysF domains, the p.Gln2001Ter mutation is situated in the C-terminal region (C2G domain) of the dysferlin protein [51]. This mutation changes the glutamine at Position 2001 to a premature stop codon, resulting in a truncated protein that lacks part of C2G domain and the entire TM helix. Deletion of the C2G domain may lead to great changes in Ca^2+^ release and a corresponding reduction in membrane repair activity [46]. The finding also suggests that the C2G subdomain contributes to membrane-binding activity [52]. On the other hand, dysferlin homodimerization is essential for its function. Dysferlin assembles into a parallel homodimer through physical interactions of C2B–C2G and the TM domains [53, 54]. The previous study indicated that truncated forms of C2 domains are not recruited to PI(4,5)P_2_ vacuoles and fail to trigger membrane tubulation. Disruption of dysferlin-dependent membrane tubulation is implicated in dysferlin-related muscle pathology [55]. Overall, it seems that the C2G-TM truncated protein produced as a result of p.Gln2001Ter mutation is incapable of dimerization and membrane anchoring, failing to interact with its partners such as caveolin-3 and annexins. Consequently, this mutation leads to the loss of protein functionality, resulting in dysferlinopathy.

The proband and his two affected sisters displayed a homozygous genotype for the p.Gln2001Ter variant. Phenotypic heterogeneity was observed among the family members. The severity of symptoms was significant in the proband, who experienced disease onset at 17 years old. His symptoms began with weakness in the lower limbs, particularly affecting his ability to rise from the ground and climb stairs. Eventually, his walking ability deteriorated, and he required a wheelchair for mobility. The two affected sisters had an onset of symptoms at Ages 14 and 19, respectively. Their symptoms were milder and characterized by lower limb weakness primarily related to difficulty standing up from the ground and climbing stairs. We found no relationship between phenotype and age of onset and phenotype. This heterogeneity is likely related to sex or modifying genes. The consanguineous parents both carry one copy of the p.Gln2001Ter variant, demonstrating the autosomal recessive inheritance of MD. With the identification of a mutation in the *DYSF* gene, the possibility of LGMDR2 or Miyoshi myopathy Type 1 was considered in the proband. Upon further detailed examination and based on previous evidence, the physician suggested LGMD for this family, particularly given that the disorder onset in the proband and his two affected sisters originated in the proximal limbs.

As previously mentioned, the proband's condition was initially diagnosed as “polymyositis” based on the result of a muscle biopsy. Polymyositis is a subtype of idiopathic inflammatory myopathies. Its main clinical features include progressive symmetric, primarily proximal muscle weakness, elevated CK levels, and inflammatory infiltrates observed in muscle biopsy. The standard treatment for polymyositis is immunosuppression [56]. Given that polymyositis shares phenotypic similarities with other types of myopathy, such as LGMD, the initial diagnosis in the proband was incorrect. As a result, the proband was treated with immunosuppressive drugs, including prednisolone, for 3 months. However, his condition worsened, prompting a re-evaluation in the neurology department. To accurately determine the type of myopathy and identify the involved genes, the family was referred to the Genetic Diagnosis Center. WES was performed, and the results revealed a pathogenic variant, p.Gln2001Ter, in the *DYSF* gene. Since mutations in the *DYSF* gene are associated with both LGMDR2 and Miyoshi myopathy Type 1 in genetic databases, a more detailed clinical assessment of the proband and his siblings was subsequently performed. This led to a final diagnosis of LGMD. This highlights the critical importance and challenges of making an accurate diagnosis in clinically heterogeneous disorders, due to overlapping symptoms and involvement of similar genes. Misdiagnosis can delay appropriate treatment, and, more concerningly, incorrect therapies may exacerbate the disease progression. Although, currently, there is no specific targeted treatment for most genetic diseases, early diagnosis and intervention are necessary in slowing disease progression and improving the survival rates and quality of life for both patients and their families. Consequently, it is vital to conduct molecular diagnostic procedures at the earliest stage of the disease to confirm the diagnosis. This can lead to prompt intervention and continuous monitoring, ultimately improving the prognosis and long-term survival of the affected individuals. Although there is currently no specific targeted treatment for most genetic disorders, early diagnosis and intervention are essential for slowing disease progression and improving survival and quality of life for patients and their families. Therefore, it is crucial to perform molecular diagnostic procedures at the earliest possible stage of the disease to confirm the diagnosis. This facilitates timely intervention, enables continuous monitoring, and ultimately improves prognosis and long-term outcomes for affected individuals.

## 5. Conclusion

This study employed WES to enhance disease diagnosis following an initial misdiagnosis. WES findings revealed a homozygous nonsense mutation in the *DYSF* gene of a 27-year-old male patient, whose clinical symptoms were consistent with LGMDR2. The research emphasizes the promising advantages of WES, including improved diagnostic accuracy, increased clinical utility, and greater time and cost efficiency compared to conventional diagnostic methods. This innovative approach may facilitate the identification of genetic variants linked to rare and diverse disorders and contribute to the progress of precision medicine. However, a limitation of this study is the absence of western blotting data to validate the WES finding and the structural modeling analysis. Furthermore, verifying the presence of mRNA would be useful to determine whether the mutation leads to mRNA degradation via nonsense-mediated decay (NMD).

## Data Availability

The data that supports the findings of this study are available in the supporting information of this article.

## Supporting Information

Supporting Information Additional supporting information can be found online in the Supporting Information section. Figure S1: The sequence of the variant analyzed in this study (NM_001130987.2). The corresponding sequences are accessible via the following links: gene, https://www.ncbi.nlm.nih.gov/nuccore/NM_001130987.2, and protein, https://www.ncbi.nlm.nih.gov/protein/195976779.
