# Supplementary material for: Whole Exome Sequencing Identified a Stop-Gained Mutation in DYSF Gene Associated With Dysferlinopathy in an Iranian Family
Source: Int J Genomics. 2025 Jul 23;2025:9103068. doi: 10.1155/ijog/9103068 (PMC12310322; doi:10.1155/ijog/9103068)
Supplement: Supporting Information — Additional supporting information can be found online in the Supporting Information section. Figure S1: The sequence of the variant analyzed in this study (NM_001130987.2). The corresponding sequences are accessible via the following links: gene, https://www.ncbi.nlm.nih.gov/nuccore/NM_001130987.2, and protein, https://www.ncbi.nlm.nih.gov/protein/195976779. [file 9103068.f1.docx]

**Identification of a Novel Stop-Gained Mutation in the *DYSF* Gene by Whole Exome Sequencing Linked to Dysferlinopathy in an Iranian Family**

**Supplementary data:**

**Figure S1:** The sequence of the variant (NM_001130987.2) analyzed in this study:


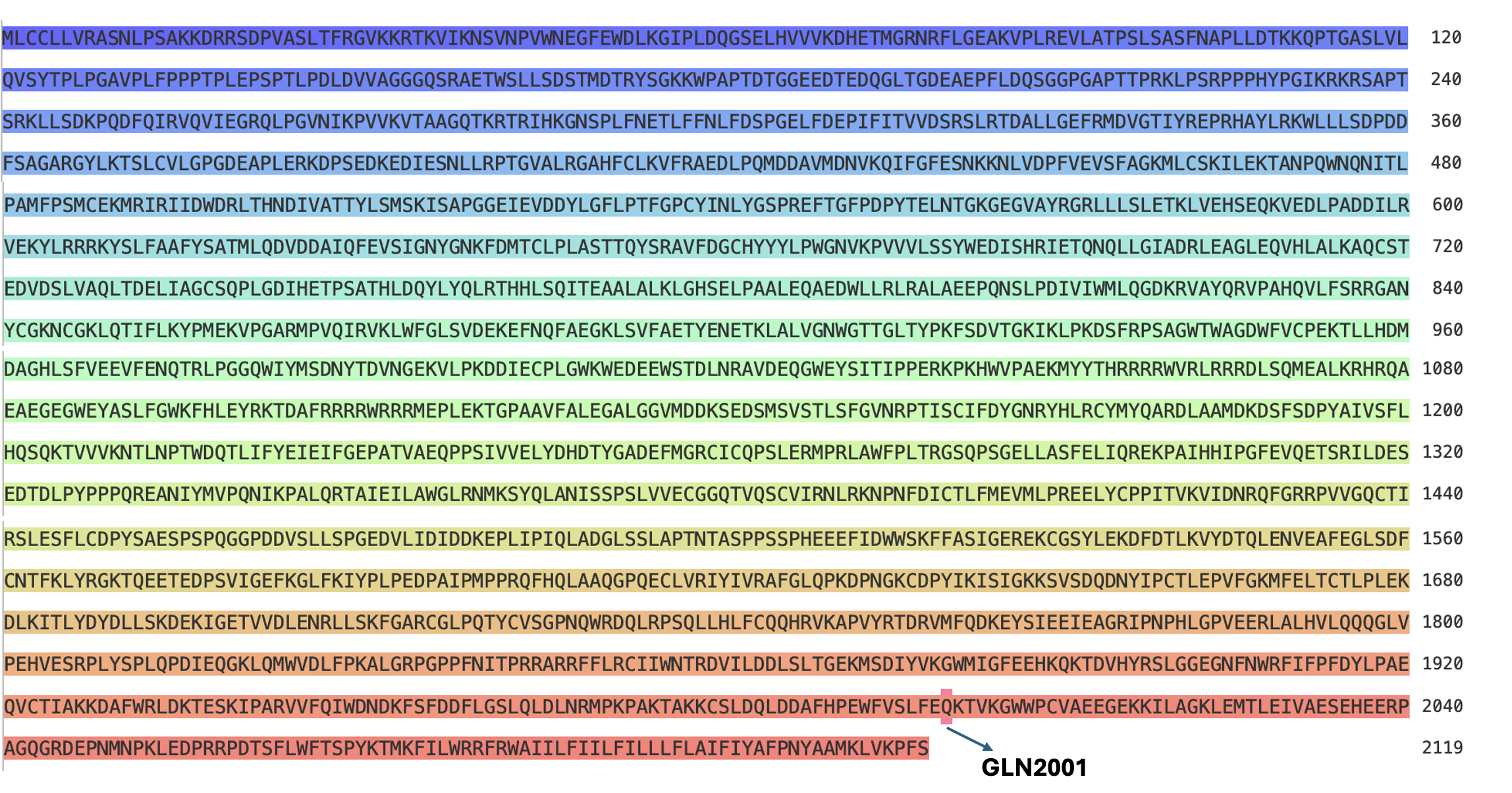


**Figure S1:** The variant analyzed in this study (NM_001130987.2).The corresponding sequences are accessible via the following links: Gene: <https://www.ncbi.nlm.nih.gov/nuccore/NM_001130987.2>, and Protein: <https://www.ncbi.nlm.nih.gov/protein/195976779>
